# Restored nitric oxide bioavailability reduces the severity of acute-to-chronic transition in a mouse model of aristolochic acid nephropathy

**DOI:** 10.1371/journal.pone.0183604

**Published:** 2017-08-23

**Authors:** Inès Jadot, Vanessa Colombaro, Blanche Martin, Isabelle Habsch, Olivia Botton, Joëlle Nortier, Anne-Emilie Declèves, Nathalie Caron

**Affiliations:** 1 Molecular Physiology Research Unit — URPhyM, NARILIS (Namur Research Institute for Life Sciences), University of Namur (UNamur), Namur, Belgium; 2 Nephrology Department, Erasme Academic Hospital and Laboratory of Experimental Nephrology, Faculty of Medicine, Université Libre de Bruxelles (ULB), Brussels, Belgium; 3 Laboratory of Molecular Biology, Faculty of Medicine and Pharmacy, Research Institute for Health Sciences and Technology, University of Mons (UMONS), Mons, Belgium; University of Louisville, UNITED STATES

## Abstract

Aristolochic Acid (AA) nephropathy (AAN) is a progressive tubulointerstitial nephritis characterized by an early phase of acute kidney injury (AKI) leading to chronic kidney disease (CKD). The reduced nitric oxide (NO) bioavailability reported in AAN might contribute to renal function impairment and progression of the disease. We previously demonstrated that L-arginine (L-Arg) supplementation is protective in AA-induced AKI. Since the severity of AKI may be considered a strong predictor of progression to CKD, the present study aims to assess the potential benefit of L-Arg supplementation during the transition from the acute phase to the chronic phase of AAN. C57BL/6J male mice were randomly subjected to daily i.p. injections of vehicle or AA for 4 days. To determine whether renal AA-induced injuries were linked to reduced NO production, L-Arg was added to drinking water from 7 days before starting i.p. injections, until the end of the protocol. Mice were euthanized 5, 10 and 20 days after vehicle or AA administration. AA-treated mice displayed marked renal injury and reduced NO bioavailability, while histopathological features of AAN were reproduced, including interstitial cell infiltration and tubulointerstitial fibrosis. L-Arg treatment restored renal NO bioavailability and reduced the severity of AA-induced injury, inflammation and fibrosis. We concluded that reduced renal NO bioavailability contributes to the processes underlying AAN. Furthermore, L-Arg shows nephroprotective effects by decreasing the severity of acute-to-chronic transition in experimental AAN and might represent a potential therapeutic tool in the future.

## Introduction

Aristolochic acid (AA) nephropathy (AAN) is a progressive tubulointerstitial nephritis of toxic origin that leads to end-stage renal disease (ESRD). The term AAN includes any form of toxic interstitial nephropathy that is caused either by ingestion of plants containing AA as part of traditional phytotherapies (known as “Chinese herbs nephropathy”), or by environmental contaminants in food (Balkan endemic nephropathy)[1–3]. Although products containing AA have been banned in most countries, AAN cases remain regularly reported all over the world and its incidence is probably highly underestimated given the weak awareness of the disease[1]. Clinically, AAN is characterized by a progressive atrophy of proximal tubules and a typical corticomedullary gradient of interstitial fibrosis[4]. Moreover, a strong correlation between AA intoxication and urothelial carcinoma has been described[5] and AA are now classified among the highly human carcinogenic substances by the World Health Organization International Agency for Research on Cancer[6].

These past decades, AAN animal models have been developed by our group[7–10]. A biphasic evolution of renal function and morphological alterations has been demonstrated[8]. First, an early phase of acute kidney injury (AKI) occurred with necrosis of proximal tubular epithelial cells (PTEC), leading to a later phase of chronic kidney disease (CKD) characterized by development of progressive interstitial fibrosis[8] with interstitial inflammation[11,12]. Moreover, defective activation of antioxidative enzymes[13,14], mitochondrial damage[15], fibroblast activation[9] and hypoxic renal injury[16–18] were demonstrated to play a crucial role in AAN physiopathology. However, the mechanisms underlying AAN progression still require investigation in order to develop effective therapeutic strategies.

Nitric oxide (NO) is known to be a key regulator in several physiological processes. Particularly in the kidney, NO is involved in many mechanisms such as the regulation of renal hemodynamic and the maintenance of sodium and water homeostasis[19]. Decreased NO bioavailability has been demonstrated in several renal disease that could contribute to progression in chronic stage[20]. We recently demonstrated that NO bioavailability was reduced in a mouse model of AA-induced AKI. Moreover, L-arginine (L-Arg) supplementation allowed to restore NO bioavailability which, in turn, improved renal function, reduced oxidative stress, prevented apoptosis and preserved tubular integrity[10]. Since the severity of AKI appears to be a strong predictor of CKD progression[21,22], we sought to evaluate the impact of L-Arg supplementation during the progression of AAN using our mouse model that has now been described as a useful tool considering the gradual progression from acute to chronic injury[23]. Indeed, NO might act as a key factor on both renal function and fibrosis development[20].

## Materials and methods

### Experimental protocol

The study conformed to APS’s guiding principles in the Care and Use of Animals and was approved by the Animal Ethics Committee of the University of Namur (approval number: 15247). Experiments were performed on 8 weeks-old C57Bl/6J male mice (Janvier, France). Mice were randomly subjected to daily intraperitoneal (i.p.) injection of either vehicle solution or aristolochic acid I (AAI) solution (3,5 mg/Kg b.w.; Sigma-Aldrich, USA) for 4 days. L-Arginine (5%; Sigma-Aldrich, USA) was supplemented in drinking water 7 days before the beginning of either vehicle or AAI of i.p. injections, until the end of the experimental protocol. The estimated dose of L-Arg was about 12 mg/g b.w. per day. Experimental groups were established as followed:

- Mice from CTL group (n = 24) received daily i.p. injections of vehicle solution;
- Mice from L-Arg group (n = 24) received daily i.p. injections of vehicle solution and L-Arg in drinking water;
- Mice from AA group (n = 24) received daily i.p. injections of AAI solution;
- Mice from AA+L-Arg group (n = 24) received daily i.p. injections of AAI solution and L-Arg in drinking water.

Body weight was measured daily in order to assess health of the mice and to adjust the drug dosages. In each group, following a 24-h period in a metabolic cage to collect urine, mice were anesthetized with a solution of ketamine (Nimatek^®^, Eurovet Animal Health, Netherlands, 80 mg/Kg b.w.) and medetomidine (Domitor^®^, Orion Pharma, Finland, 0.5 mg/Kg b.w.) and then killed by intracardiac puncture and therefore exsanguination either at 5 (n = 8), 10 (n = 8) or 20 days (n = 8) after the first i.p. injection of vehicle or AAI. Blood sample was collected by cardiac puncture, both kidneys were excised and immediately processed for further analyses. In order to avoid the multiplication of figures in this manuscript, we decided to not illustrate L-Arg group since L-Arg impact on control mice as already been demonstrated by our group in a previous study [10]. Indeed, in control condition, L-Arg treatment had no impact on all parameters except for urinary NOx level that was significantly increased at Day 5 and Day 20 compared to control mice. A significant increase in urinary cGMP level at Day 10 compared to control mice was also demonstrated. In contrast, *eNOS*, *iNOS* and *nNOS* mRNA expressions were unchanged with L -Arg treatment in control mice compared to the control group without L -Arg treatment (S1 Fig).

### Determination of NO bioavailability

Concentrations of NO_2_/NO_3_ metabolites and cGMP were measured in urine samples using colorimetric assays (Cayman Chemical Company, USA) as previously described [10].

### Measurement of urinary and plasma markers

Blood samples were centrifuged at high speed for 10 min at 4°C. Plasma was collected and frozen at − 80°C until use. Plasma creatinine concentration was determined using a sensitive accurate HPLC method (Spherisorb5-μm SCX column, 4.0 × 250 mm; Waters, USA). Blood urea nitrogen was measured using a colorimetric assay (BioAssay Systems, USA) following the manufacturer’s protocol. Urinary protein levels were assayed according to the Bradford method using bovine serum albumin as a standard.

### Renal histology

#### Periodic acid–Schiff staining (PAS)

Tissue samples were fixed in Duboscq-Brazil fluid and embedded in paraffin. Five-μm sections were prepared and exposed to Schiff’s reagent to perform PAS staining in order to highlight renal damages. Tubular injury scoring was assessed on kidney sections. For each sample, 10 consecutive fields were analysed at a magnification of 200× and were considered positive for injury based on the presence of tubular necrosis or atrophy, luminal casts and alterations in the brush border. Tubular injury was expressed as a percentage.

#### Sirius Red staining

To assess collagen I and III deposits, 5-μm sections were stained with picrosirius red (Sirius Red).

### Immunohistochemistry

Immunohistochemical detection of macrophages, lymphocytes and myofibroblasts (using α-SMA) was performed on paraffin-embedded kidney sections. Briefly, after dewaxing and rehydration, a microwave pre-treatment in citrate buffer (pH 6.2) was performed to unmask antigens present in the renal tissue. Tissue sections were then incubated for 1 h with primary antibodies: anti-macrophages (rat anti-mouse F4/80 antibody, Abcam, UK), anti-lymphocytes (rabbit anti-mouse CD3 antibody, Abcam, UK) and anti-α-SMA (rabbit anti-mouse antibody, Abcam, UK). After rinsing in PBS, slides were exposed for 30 min to the appropriate secondary antibody. Kidney sections were finally incubated with ABC complex (Vector Laboratories, UK) for 30 min and bound peroxidase activity was detected with the DAB kit (DAKO, Belgium). Counterstaining was performed with hemalun and Luxol fast blue.

### Morphometric analysis in the renal tissue

The relative area occupied by α-SMA staining and Sirius Red in the renal tissue was evaluated by a computer-assisted morphometric approach as previously described [24]. Evaluation of the relative positive area was performed on one section per experimental animal. For each section, 10 square fields (0.084 mm^2^/field) were observed at 400x magnification in each renal zone. The relative area occupied by the positive staining was expressed as a percentage.

### Cell counts

The frequency of macrophage-positive cells and lymphocyte-positive cells in the interstitium was evaluated by a semi-quantitative analysis. Briefly, the distribution of positive cells was performed on one section per experimental animal. For each section, 10 square fields (0.084 mm^2^/field) were observed at 400× magnification. Quantifications were performed by two investigators blind to the group origin of the mouse.

### Quantitative real-time PCR

Frozen kidneys were homogenized and total RNA was then extracted with Trizol (Sigma-Aldrich, USA) and treated with DNAse (Promega, Belgium). Total RNA concentration was measured by NanoDrop (NanoDrop 1000, Thermo Scientific, USA). Transcript-specific primers were generated on the basis of mouse sequences from GenBank. NCBI Primer Blast was used to ensure specificity of primers for each target. All pairs of primers were analysed for dissociation curves and melting temperatures. Real-time quantitative PCR was performed in order to quantify mRNA level of *eNOS*, *iNOS*, *nNOS*, *IL-1β*, *IL-6*, *TNFα*, *Col I*, *Col III*, *CTGF*, *Periostin*, *TGFβ*, *Smad3* and *18S* as a housekeeping gene (see Table 1). Briefly, 2 μg of total RNA were used for reverse transcription using MLV reverse transcriptase (Promega, Belgium) during 1 h at 70°C. Quantitative PCR amplification was performed using the SYBR Green Master Mix (Roche, Belgium) and the Prism 7300 Real-Time PCR Detection System (Applied Biosystems, CA, USA). Mean fold changes were calculated by averaging the duplicate measurement for each gene. Relative gene expressions were calculated using the 2^−ΔΔCT^ method.

**Table 1:** Sequences of the primers used for qRT-PCR.

Gene		Primer Sequences (5'-3')
**eNOS**	F	AACCATTCTGTATGGCTCTGAGAC
	R	CTCTAGGGACACCACATCATACTC
**iNOS**	F	CAGCTGGGCTGTACAAACCTT
	R	ATGTGATGTTTGCTTCGGACA
**nNOS**	F	ACCCTGTGCGAGATCTTCAA
	R	TGGTGACCACCAAGACCAG
**NGAL**	F	ATGTCACCTCCATCCTGGTC
	R	CCTGTGCATATTTCCCAGAGT
**IL-1β**	F	AGTTGACGGACCCCAAAAG
	R	AGCTGGATGCTCTCATCAGG
**IL-6**	F	GCTACCAAACTGGATATAATCAGGA
	R	CCAGGTAGCTATGGTACTCCAGAA
**TNFα**	F	TACTGAACTTCGGGGTGATTGGTCC
	R	CAGCCTTGTCCCTTGAAGAGAACC
**Col I**	F	GCAGGTTCACCTACTCTGTCCT
	R	CTTGCCCCATTCATTTGTCT
**Col III**	F	TCCCCTGGAATCTGTGAATC
	R	TGAGTCGAATTGGGGAGAAT
**CTGF**	F	TGACCTGGAGGAAAACATTAAGA
	R	AGCCCTGTATGTCTTCACACTG
**Periostin**	F	TCGTGGAACCAAAAATTAAAGTC
	R	CTTCGTCATTGCAGGTCCTT
**TGFβ**	F	TGGAGCAACATGTGGAACTC
	R	GTCAGCAGCCGGTTACCA
**Smad3**	F	GCCACTGTCTGCAAGATCC
	R	AGCTAGGAGGGCAGCAAAT
**18S**	F	CGCCGCTAGAGGTGAAATTCT
	R	CGAACCTCCGACTTTCGTTCT

### Statistics

Results are presented as mean values ± SEM. Statistical group analysis was performed with SigmaStat 11.0 (Systat Software, San Jose, CA, USA). Comparisons between groups were evaluated by two-way ANOVA. Significant analysis of variance was followed by the Holm-Sidak multiple-comparisons procedure. A P-value < 0.05 was considered statistically significant.

## Results

### L-arginine supplementation prevents the decrease in NO bioavailability

**Fig 1:**
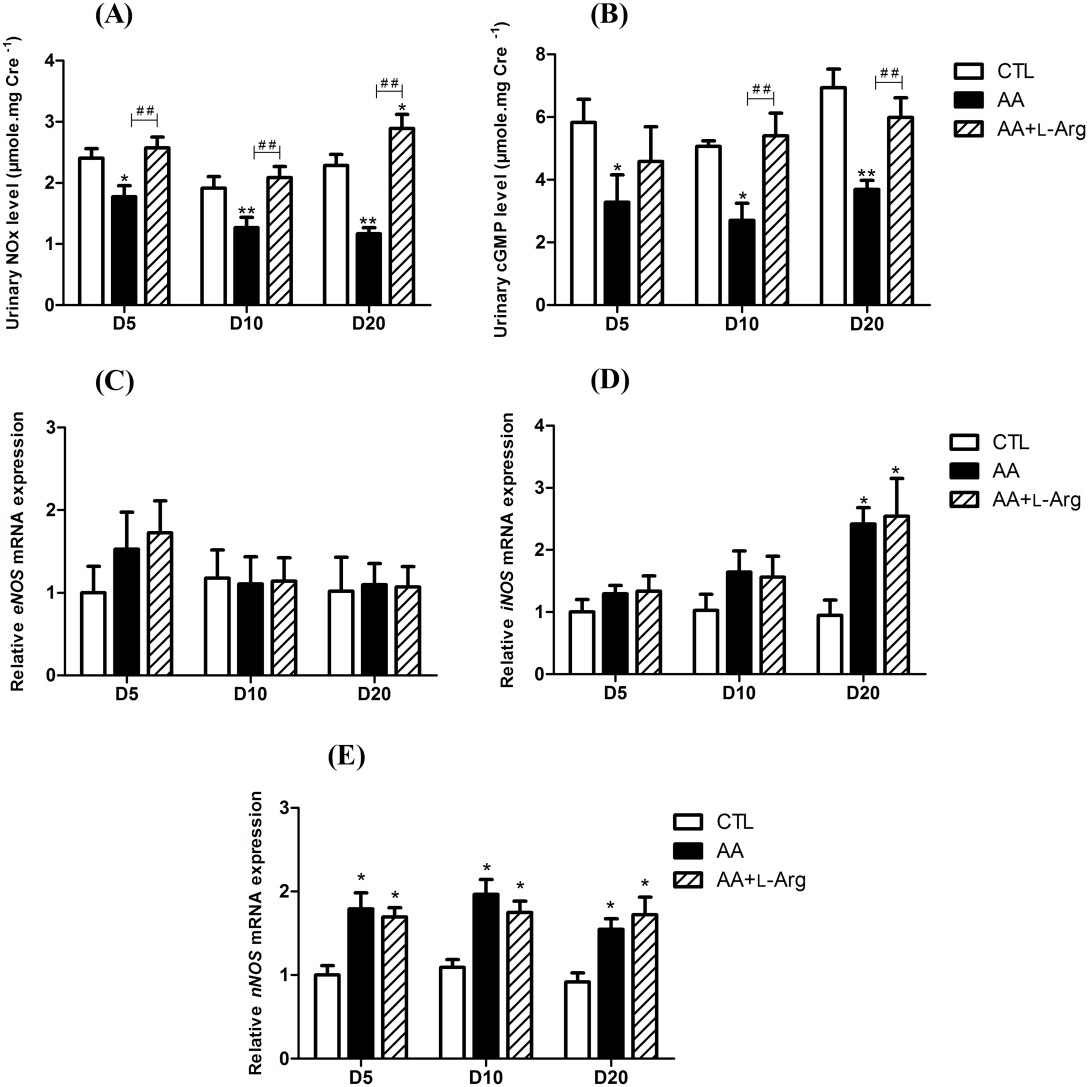
Urinary NOx and cGMP excretions at days 5, 10 and 20 in CTL, AA and AA+L-Arg mice and relative kidney expression of nitric oxide synthases mRNA (2^-Δ ΔCT^) at days 5, 10 and 20 in CTL, AA and AA+L-Arg mice. (**A**) Urinary nitrite/nitrate (NOx) concentrations in CTL, AA and AA+L-Arg mice. (**B**) Urinary cGMP concentrations in CTL, AA and AA+L-Arg mice. Real-time quantitative PCR for *endothelial nitric oxide synthase* (*eNOS*) (**C**), *inducible nitric oxide synthase* (*iNOS*) (**D**) and *neuronal nitric oxide synthase* (*nNOS*) (**E**) mRNA expression was performed on kidney tissue from CTL, AA and AA+L-Arg mice normalized against *18S*. Data are presented as means ± SEM; *n* = 8 in each group. Statistical analysis were performed by two-way ANOVA followed by Holm-Sidak test. *P ≤ 0,05 *vs* CTL mice, **P ≤ 0,01 *vs* CTL mice, ^##^ *P* ≤ 0,01 *vs* AA mice.

As shown in Fig 1A and 1B, AA treatment induced a significant decrease in urinary concentrations of NO metabolites (NO_x_) and cGMP throughout the experimental protocol, that were prevented by L-Arg treatment. Intrarenal expression of *eNOS*, *iNOS* and *nNOS* was measured in all group of mice, using quantitative RT-PCR analysis. Renal expression of *eNOS* mRNA (Fig 1C) in mice treated either with AA or with AA and L-Arg did not differ compared to CTL mice. An increase of *iNOS* mRNA (Fig 1D) expression was observed at day 20 in AA treated mice (2.42 ± 0.26, *P*<0.009) as well as in AA+L-Arg mice (2.54 ± 0.61, *P*<0.005) compared to CTL (0.94 ± 0.25). A slight increase of *nNOS* mRNA (Fig 1E) expression was also observed in AA mice as well as in AA+L-Arg mice all along the protocol.

### Effect of L-arginine supplementation on renal function

To better characterize the effect of L-Arg supplementation on renal function in AA-treated mice, urinary and plasma markers of renal function were measured. As shown in Table 2, AA mice displayed polyuria at day 10 (1.35 ± 0.17 ml.24h^-1^, *P*<0.012) and at day 20 (2.02 ± 0.28 ml.24h^-1^, *P*<0.001) after AA intoxication compared to CTL mice (0.59 ± 0.13 ml.24h^-1^ and 0.79 ± 0.20 ml.24h^-1^, respectively). In L-Arg-treated mice, polyuria was prevented at day 10 (0.67 ± 0.13 ml.24h^-1^, *P*<0.013). At day 20, even though the polyuria was significantly lower than in mice only treated with AA (*P*<0.023), it remains still significantly higher compared to CTL mice (1.43 ± 0.19 ml.24h^-1^, *P*<0.028). AA intoxication also induced an increase in plasma creatinine (PCr) and blood urea nitrogen (BUN) that reached a maximum level at day 10. These changes were partially prevented by L-Arg supplementation at this timepoint.

**Table 2:** Effect of L-arginine supplementation on renal function at days 5, 10 and 20 in CTL, AA and AA+L-Arg mice.

	Diuresis–ml.24h^-1^	Creatinine clearance–ml.min^-1^	Plasma creatinine level– μmol.l^-1^	Blood urea nitrogen– μmol.l^-1^	Proteinuria–mg.mg Cre^-1^
**Day 5**					
CTL	0.43 ± 0.10	1.29 ± 0.10	3.51 ± 0.27	11.31 ± 1.15	5.31 ± 0.18
AA	0.45 ± 0.21	0.15 ± 0.02 ***	12.01 ± 2.28	9.16 ± 1.44	16.09 ± 3.71 ***
AA + L-Arg	0.45 ± 0.12	1.12 ± 0.35 ^##^	5.95 ± 1.31	7.55 ± 1.90	10.41 ± 2.51
**Day 10**					
CTL	0.59 ± 0.13	1.02 ± 0.16	4.02 ± 0.63	11.07 ± 1.46	5.45 ± 1.02
AA	1.35 ± 0.17 *	0.29 ± 0.09 ***	27.29 ± 5.25 ***	183.10 ± 41.72 ***	14.28 ± 2.67 **
AA + L-Arg	0.67 ± 0.13 ^#^	1.41 ± 0.43 ^###^	3.72 ± 1.01 ^###^	100.80 ± 30.81 * ^##^	12.08 ± 1.34 *
**Day 20**					
CTL	0.79 ± 0.20	1.11 ± 0.19	3.91 ± 0.63	11.65 ± 1.42	6.92 ± 0.37
AA	2.02 ± 0.28 ***	0.57 ± 0.11	17.06 ± 3.11 **	100.80 ± 18.60	11.01 ± 0.96
AA + L-Arg	1.43 ± 0.19 * ^#^	0.47 ± 0.04	12.52 ± 1.56	111.30 ± 17.10	11.87 ± 0.51

Statistical analysis was performed using two-way ANOVA followed by Holm-Sidak test. Data are presented as means ± SEM; *n* = 8 in each group.

*P ≤ 0,05 *vs* CTL mice,

**P ≤ 0,01 *vs* CTL mice,

***P ≤ 0,001 *vs* CTL mice,

^#^*P* ≤ 0,05 *vs* AA mice,

^##^*P* ≤ 0,01 *vs* AA mice

^###^*P* ≤ 0,001 *vs* AA mice.

### L-arginine supplementation reduces tubular injury

**Fig 2:**
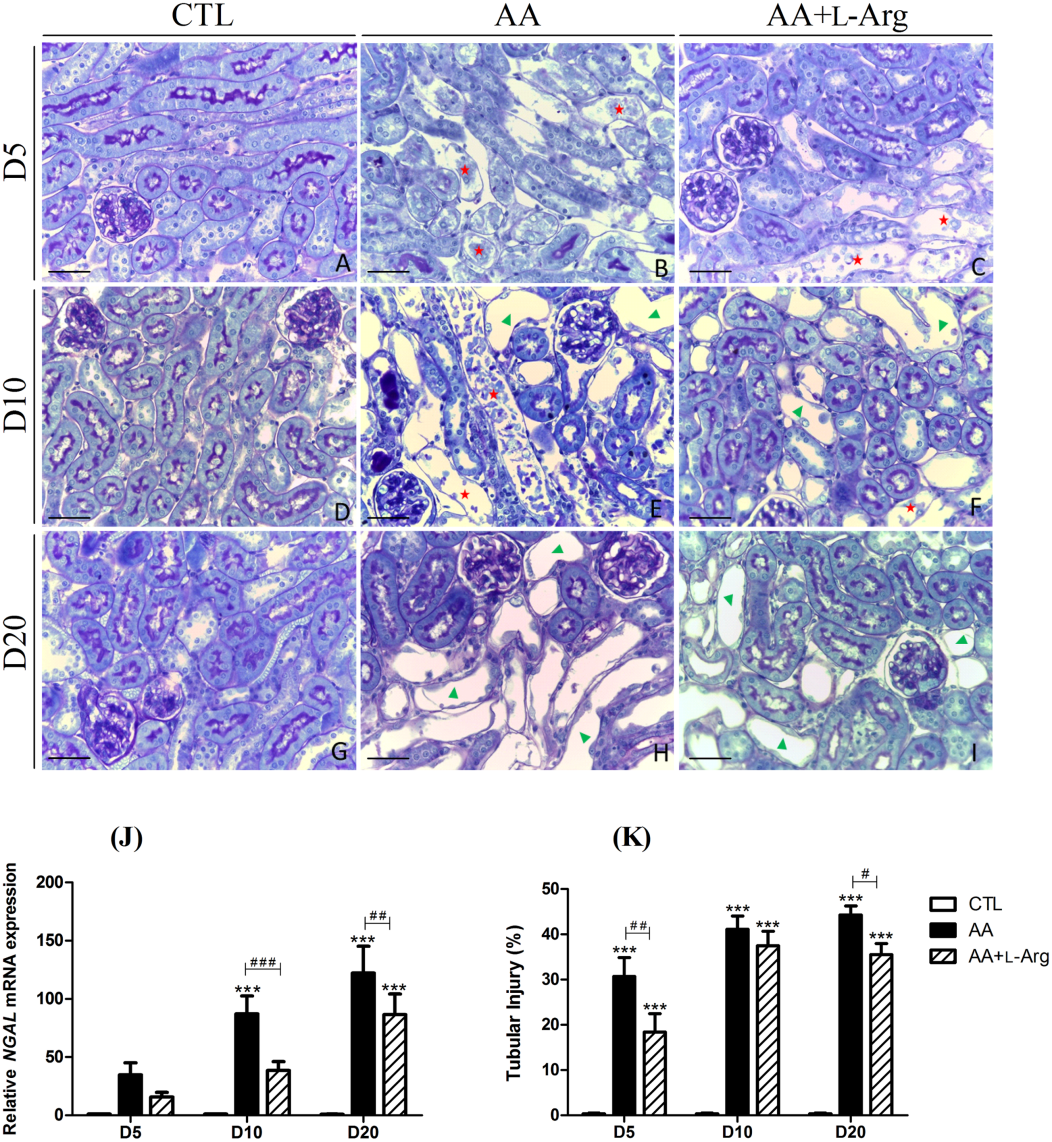
Effect of L-arginine supplementation on tubular injury score and on relative kidney expression of Neutrophil Gelatinase-Associated Lipocalin (*NGAL*) mRNA at days 5, 10 and 20 in CTL, AA and AA+L-Arg mice. Tubular injury in CTL, AA and AA+L-Arg mice. Representative photographs of hemalun, Luxol fast blue and Periodic Acid Schiff stained kidney sections (x400) from CTL (**A,D,G**), AA (**B,E,H**) and AA+L-Arg (**C,F,I**) mice at days 5, 10 and 20. Necrotic tubules (red) with cell debris in tubular lumens are visible in AA and AA+L-Arg treated mice at days 5 and 10 and cystic tubules (green) are visible in AA and AA+L-Arg treated mice at days 10 and 20. Scale bar = 50 μm. Real-time quantitative PCR for *Neutrophil Gelatinase-Associated Lipocalin* (*NGAL*) mRNA expression was performed with kidney tissue from CTL, AA and AA+L-Arg mice normalized against *18S* (**J**). Quantitative analysis of tubular injury in CTL, AA and AA+L-Arg mice at days 5, 10 and 20 (**K**). Statistical analysis was performed using two-way ANOVA followed by Holm-Sidak test. Data are presented as means ± SEM; *n* = 8 in each group. ***P ≤ 0,001 *vs* CTL mice, ^#^*P* ≤ 0,05 *vs* AA mice, ^##^*P* ≤ 0,05 *vs* AA mice and ^###^*P* ≤ 0,001 *vs* AA mice.

To characterize the effect of L-Arg supplementation on tubular injury, a morphological analysis was performed along with the measurement of *NGAL* mRNA expression, a marker of tubular injury. As illustrated in Fig 2, CTL mice displayed normal renal structures (Fig 2A, 2D and 2G). At day 5 (Fig 2B), AA treatment induced necrosis of PTEC, tubular cell detachment, with the presence of cell debris in tubular lumens in the outer stripe of outer medulla (OSOM), with some extension to the cortex. A similar pattern was observed in AA+L-Arg mice but at a lesser extend as attested by the decreased tubular injury percentage (30 ± 3% for AA mice and 18 ± 3% for AA+L-Arg mice, *P*<0.006). Histological analysis of kidney sections from AA and AA+L-Arg at day 10 (Fig 2E and 2F) revealed appearance of atrophic tubules along with persistence of necrotic tubule area. At that time-point, L-Arg treatment did not significantly decrease the tubular injury score (Fig 2K). Finally, at day 20, numerous atrophic tubules were detected in AA-treated mice and AA+L-Arg-treated mice. Interestingly, L-Arg-treated mice presented a slight decrease of tubular injury (44 ± 2% for AA mice and 36 ± 2% for AA+L-Arg mice, *P*<0.032). The results of this morphological analysis were correlated with *NGAL* mRNA expression. Indeed, a significant increase of mRNA expression levels of *NGAL* was observed in AA-treated mice at day 10 and day 20 of the protocol that was attenuated at both time-points with L-Arg supplementation (Fig 2J).

### L-arginine supplementation reduces inflammation

**Fig 3:**
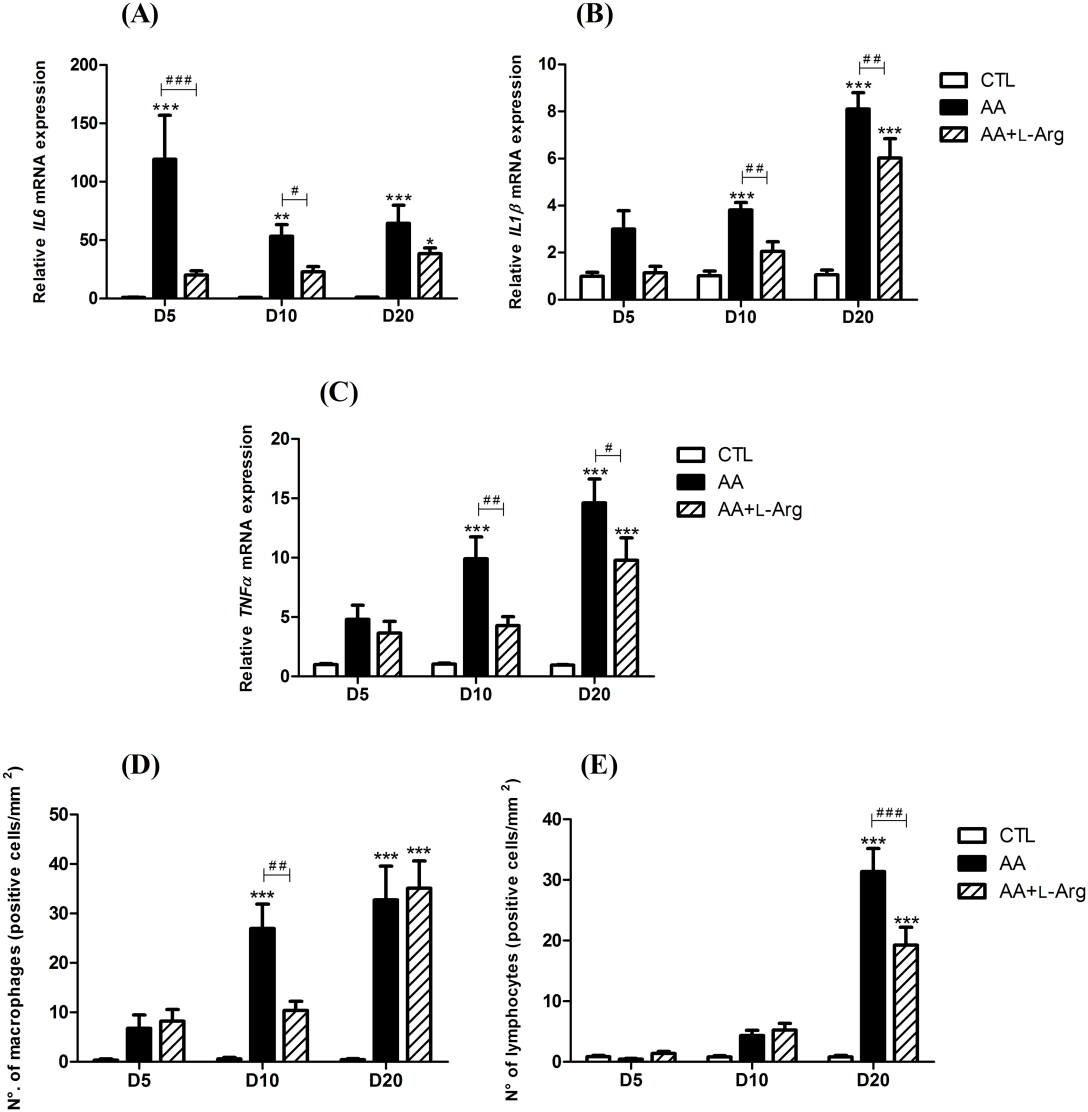
Effect of L-arginine supplementation on relative kidney expression of inflammatory cytokines; interleukin 6 (*IL6*), interleukin 1β (*IL1β*) and tumor necrosis factor α (*TNFα*) mRNA (2^-Δ ΔCT^) and on on interstitial infiltration of macrophages and lymphocytes at days 5, 10 and 20 in CTL, AA and AA+L-Arg mice. Real-time quantitative PCR for *interleukin 6* (*IL6*) (**A**), *interleukin 1β* (*IL1β*) (**B**) and *tumor necrosis factor α* (*TNFα*) (**C**) mRNA expression was performed on kidney tissue from CTL, AA and AA+L-Arg mice normalized against *18S*. Macrophage (**D**) and lymphocyte (**E**) accumulation within the interstitium of kidney from CTL, AA and AA+L-Arg mice at days 5, 10 and 20. Statistical analysis was performed using two-way ANOVA followed by Holm-Sidak test. Data are presented as means ± SEM; *n* = 8 in each group. *P ≤ 0,05 *vs* CTL mice, **P ≤ 0,01 *vs* CTL mice, ***P ≤ 0,001 *vs* CTL mice, ^#^*P* ≤ 0,05 *vs* AA mice, ^##^*P* ≤ 0,01 *vs* AA mice and ^###^*P* ≤ 0,001 *vs* AA mice.

Since cytokines are critical factors determining the inflammatory response during the progression to CKD, we performed quantitative RT-PCR analysis to evaluate their involvement during the time-course of our model. As shown in Fig 3A, intrarenal *IL6* mRNA was highly upregulated throughout the protocol in AA-treated mice, with the highest over-expression measured at 5 days after AAN induction (nearly 120-fold increase relative to control). L-Arg treatment significantly reduced this upregulation at day 5 and day 10. Expression of *IL1β* mRNA (Fig 3B) was also significantly increased throughout the protocol in AA-treated mice to reach a highest level at day 20 (8.10 ± 0.69 fold increase relative to control). This upregulation was significantly reduced with L-Arg treatment at day 10 and day 20. A similar pattern was observed for mRNA expression of *TNFα* (Fig 3C) that reached 14.60 ± 2.02 fold increase relative to control at day 20. A significant reduction of *TNFα* mRNA expression was also revealed after L-Arg treatment at day 10 and day 20 in AA-treated mice. Finally, interstitial macrophages (F4/80—positive cells, Fig 3D) were accumulated in the kidney tissue of AA-treated mice throughout the protocol with a significant amount at days 10 and day 20. This process was prevented by L-Arg treatment at both time-points. In addition, significant accumulation of interstitial lymphocytes (CD3—positive cells, Fig 3E) observed at day 20 in AA-treated mice was significantly reduced by L-Arg supplementation.

### L-arginine supplementation reduces fibrosis

**Fig 4:**
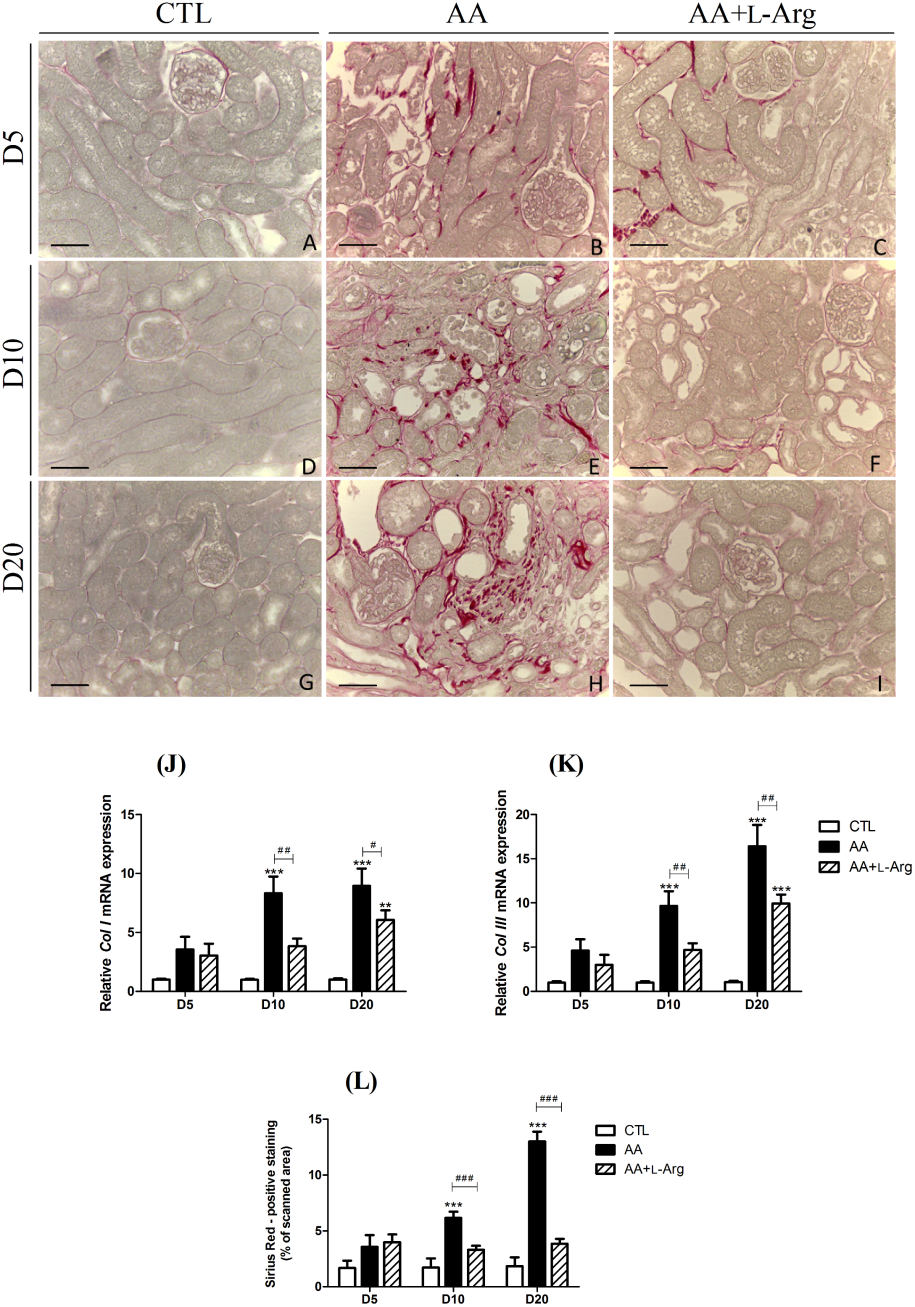
Effect of L-arginine supplementation on Sirius Red staining at days 5, 10 and 20 in CTL, AA and AA+L-Arg mice. Representative photographs of picrosirius red stained kidney sections (x400) from CTL (**A,D,G**), AA (**B,E,H**) and AA+L-Arg (**C,F,I**) mice at days 5, 10 and 20. Scale bar = 50 μm. Real-time quantitative PCR for *ColI* (**J**) and *ColIII* (**K**) mRNA expression was performed with kidney tissue from CTL, AA and AA+L-Arg mice normalized against *18S* Quantitative analysis of picrosirius red expression in CTL, AA and AA+L-Arg mice at days 5, 10 and 20 (**L**). Statistical analysis was performed using two-way ANOVA followed by Holm-Sidak test. Data are presented as means ± SEM; *n* = 8 in each group. **P ≤ 0,01 *vs* CTL mice, ***P ≤ 0,001 *vs* CTL mice, ^#^*P* ≤ 0,05 *vs* AA mice, ^##^*P* ≤ 0,01 *vs* AA mice and ^###^*P* ≤ 0,001 *vs* AA mice.

**Fig 5:**
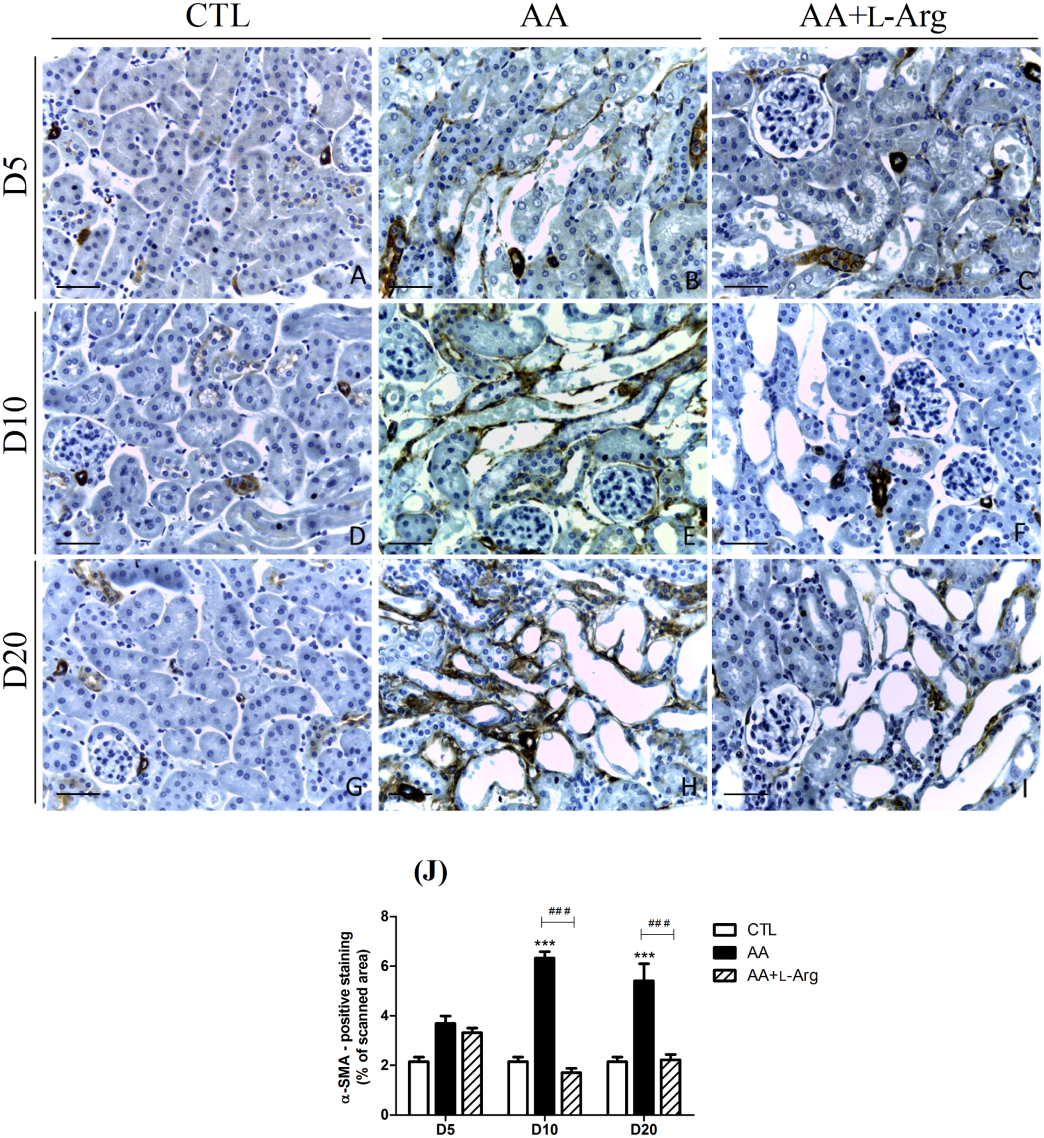
Effect of L-arginine supplementation on α-SMA expression at days 5, 10 and 20 in CTL, AA and AA+L-Arg mice. Representative photographs of α-SMA immunostained kidney sections (x400) from CTL (**A,D,G**), AA (**B,E,H**) and AA+L-Arg (**C,F,I**) mice at days 5, 10 and 20. Scale bar = 50 μm. Quantitative analysis of α-SMA expression in CTL, AA and AA+L-Arg mice at days 5, 10 and 20 (**J**). Statistical analysis was performed using two-way ANOVA followed by Holm-Sidak test. Data are presented as means ± SEM; *n* = 8 in each group. ***P ≤ 0,001 *vs* CTL mice and ^###^*P* ≤ 0,001 *vs* AA mice.

**Fig 6:**
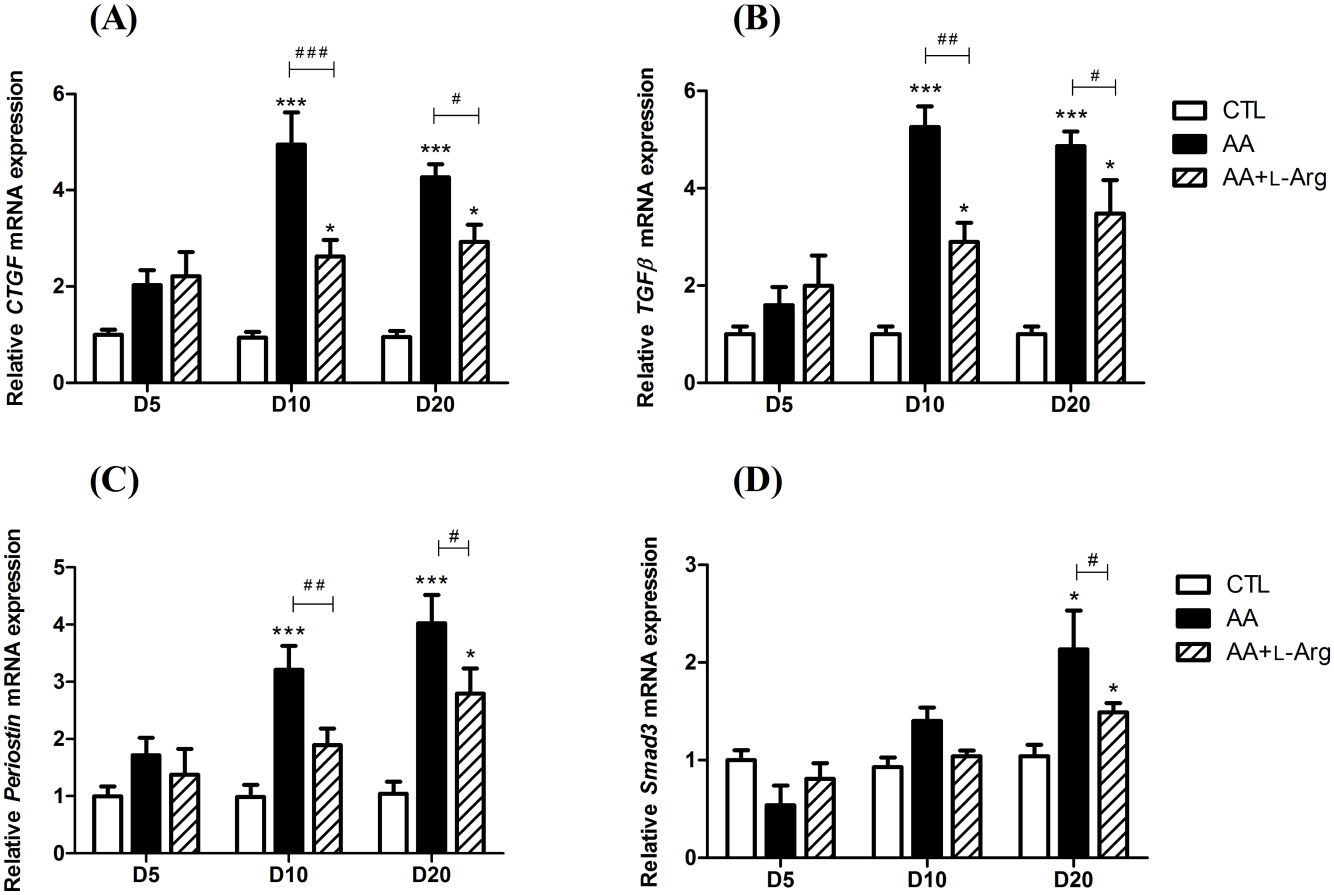
Effect of L-arginine supplementation on relative kidney expression of profibrotic factors; connective tissue growth factor (*CTGF*), transforming growth factor β (*TGFβ*), *periostin* and *Smad3* mRNA (2^-Δ ΔCT^) at days 5, 10 and 20 in CTL, AA and AA+L-Arg mice. Real-time quantitative PCR for *CTGF* (**A**), *TGFβ* (**B**), *Periostin* (**C**) and *Smad3* (**D**) mRNA expression was performed with kidney tissue from CTL, AA and AA+L-Arg mice normalized against *18S*. Statistical analysis was performed using two-way ANOVA followed by Holm-Sidak test. Data are presented as means ± SEM; *n* = 8 in each group. *P ≤ 0,05 *vs* CTL mice, ***P ≤ 0,001 *vs* CTL mice, ^#^*P* ≤ 0,05 *vs* AA mice, ^##^*P* ≤ 0,01 *vs* AA mice and ^###^*P* ≤ 0,001 *vs* AA mice.

Since progressive tubulointerstitial fibrosis is a sign of progressive ESRD, specific markers of renal fibrosis were investigated. Collagen deposition was studied by morphometric analysis in Sirius red-stained renal sections as an index of the fibrotic response. As illustrated in Fig 4B, 4E and 4H, collagen I and III were accumulated in the interstitium of AA-treated mice from day 10 and even enhanced at day 20, reflecting the progression to chronic injury. This observation was confirmed by the quantitative analysis of Sirius Red positive staining (Fig 4L) as well as by the upregulation of *Col I* and *Col III* mRNA expression (Fig 4J and 4K). L-Arg treatment significantly reduced collagen I and III deposition (Fig 4C, 4F, 4I and 4L) as well as the upregulation of *Col I* and *Col III* mRNA expression (Fig 4J and 4K). Moreover, the increase in α-SMA expression, reflecting the amount of myofibroblasts in the renal interstitium, observed at 10 and 20 days after AA intoxication was also completely prevented by L-Arg treatement (Fig 5). The quantitative RT-PCR measurement of the AA-induced expression of *CTGF*, *TGFβ*, *Periostin* and *Smad3* all considered as key profibrotic markers (Fig 6), demonstrated their significant increase with the AA treatment at day 10 and day 20. These upregulations were significantly reduced with L-Arg treatment.

## Discussion

Since the identification of AA toxicity in Belgium in 1992[25], experimental *in vitro* and *in vivo* models have been developed and studies have been undertaken in order to understand underlying molecular and cellular mechanisms of AAN pathophysiology. Up to now, the PTEC have always been reported to be the primary target of AA-induced toxicity[8,9]. However, only few studies have addressed the involvement of the vascular network and impairment of vasoactive mediators in AAN pathophysiology[4,10,16,18,26]. In this regard, Depierreux and colleagues first underlined the issue about the vascular network in AAN pathology[4]. Since then, decrease in peritubular capillaries has been described[26,27] associated to a reduced expression of vascular endothelial growth factor (VEGF) along with an increased expression of hypoxia inducible factor-1 (HIF-1). These observations suggested that ischemia and hypoxia could contribute to AAN progression[27]. Similar data, reported by Wen et al., revealed an imbalance between vasoactive factors with a reduced NO production, while expression of endothelin-1 (ET-1) was increased in a rat model of AAN[16]. Since the kidney is characterized by a high energy demand, this organ is very susceptible to further compromise of vascular perfusion and oxygenation[28]. Although it is well known that injury of renal microvasculature, endothelial cell activation as well as imbalance between vasoactive substances widely contributes to the progression from AKI to CKD[28–31], these mechanisms remain poorly investigated in AAN pathogenesis.

In the kidney, NO plays major roles in the regulation of vascular resistance, glomerular filtration rate, water and sodium excretion, and in the maintenance of renal structural integrity[19]. In most kidney diseases, renal NO level is reduced which could contribute to further progression to CKD and ESRD[20,32]. In this regard, L-Arg supplementation was found to play a protective role by improving renal function in *in vivo* models of nephrotoxicity as induced by cisplatin[33], cyclosporine[34,35] and gentamicin[36]. Conversely, chronic administration of a non-selective inhibitor of NOSs, L-NAME (N-nitro-L-Arg methyl ester) leads to worsening of renal injury in the same models[33,35,36].

In view of these elements, our group investigated for the first time NO involvement in a mouse model of AAN[10]. In this previous study, we established a reduction in NO bioavailability in mice intoxicated with AA, occurring as soon as five days after the beginning of the AA intoxication. This effect was associated with a decrease of the renal function, massive necrosis of PTEC, increased inflammation and oxidative stress. We then demonstrated that restoring renal NO bioavailability through L-Arg supplementation reduced oxidative stress, improved renal function and preserved renal structure[10]. These data may lead to consider that impairment of vasoactive mediators such as NO constitutes an early event of the disease, suggesting that impairment of the vascular compartment contributes to progression to later stage.

In the present study, we extended our investigation to the progression from AKI to CKD in our AAN model. We demonstrated for the first time that reduction in NO bioavailability plays a crucial role in AA-induced AKI-to-CKD transition. Indeed, L-Arg supplementation shows nephroprotective effects by reducing the severity of the transition from AKI to CKD and most importantly, by reducing fibrosis development.

Firstly, our results confirmed the reduction of urinary NO_x_ metabolites and cGMP levels reflecting a decreased renal NO bioavailability in our AAN model throughout the experimental protocol. L-Arg supplementation allowed to restore renal NO levels as already described by our group[10] as well as in other models of nephrotoxicity[33,35,37].

AA nephrotoxicity was demonstrated by polyuria and a marked increase in plasma creatinine and BUN. Oral administration of L-Arg to AA-treated mice resulted in complete normalization of plasma creatinine level and significantly reduced BUN level. These data support previous findings reporting similar effects in cisplatin and cyclosporine models[33–36] thereby highlighting the protective role of L-Arg supplementation. Histological examination of kidney sections confirmed the occurrence of renal damage as attested by histopathological features of AAN observed in our model. At first, tubular necrosis occurs in the early phase of AKI, leading to development of chronic lesions such as tubular atrophy and interstitial fibrosis observed in the later phase. Treatment with L-Arg improved the pathological changes induced by AA treatment as also reported in the cisplatin and cyclosporine nephrotoxicity model[33,35].

It is well established that after an episode of AKI, persistent maladaptive repair mechanisms promote inflammation. In the present study, renal mRNA expression of pro-inflammatory cytokines, *IL-6*, *IL-1β* and *TNF-α*, was increased following AA treatment as also observed in cisplatin-induced nephrotoxicity[38]. With L-Arg treatment, the mRNA upregulation of these cytokines was significantly dampened. Macrophages are key regulators of inflammation and fibrosis[39]. Although it does not prove causality, it has been reported that the number of interstitial macrophages was closely correlated with interstitial fibrosis, atrophic tubules, reduced capillary density, and decreased renal survival[28]. In our study, L-Arg supplementation reduced renal inflammatory injury as attested by decreased macrophages and lymphocytes infiltration as previously reported in the literature in a rat model of obstructive nephropathy and puromycin-induced nephrosis[40]. Moreover, it has been recently shown that different subpopulations of macrophages may play specific roles in the course from AKI to CKD[39]. These findings underscore the urgent need of understanding macrophage function and characterizing these subpopulations as well as their specific role in the pathogenesis of AAN.

The most striking feature reported in AAN is an extensive interstitial fibrosis[4]. Here, our results demonstrate that L-Arg treatment reduced collagen accumulation as already described in other models of kidney disease[37,41]. Since endothelial dysfunction may be considered as an important factor in progression of renal fibrosis, NO might play a key role by controlling extracellular matrix synthesis during fibrogenesis[42,43]. Interestingly, accumulation of asymmetric dimethylarginine (ADMA), an endogenous NOS inhibitor, in the plasma of patients suffering from CKD[20,44], has been reported and proposed to contribute to endothelial dysfunction during fibrosis development[44], thereby confirming that NO plays a central role in fibrogenesis. Indeed, NO is directly linked to the TGF-β pathway since ADMA administration in mice was associated with a strong induction of mRNA and protein expression of TGF-β[44]. In our study, we were able to confirm this crosstalk since L-Arg supplementation partially prevented AA-induced *TGF-β* mRNA upregulation as also reported in a rat model of unilateral ureteral obstruction[41]. Moreover, we also confirmed that downregulation of Smad3 protected mice from chronic AAN as reported by Zhou and collaborators [45] and that blockade of the TGF- β/Smad3 signaling pathway may have therapeutic potential for prevention of treatment of chronic AAN. We also established for the first time that *Periostin* mRNA is overexpressed in AAN. This result is not surprising since, in addition of being a marker of progression of renal fibrosis, periostin has been reported to mediate renal disease through interaction with the TGF-β pathway[46]. This effect of periostin might therefore explain the similar pattern of *TGF-β* and *Periostin* mRNA expression observed throughout our protocol. Finally, the degree of fibrotic response is strongly linked to the degree of the inflammatory response. We can therefore postulate that some beneficial effects of NO on reducing renal fibrosis are related to attenuation of inflammatory cells infiltration as discussed in the previous section (S2 Fig).

Based on our results, we reported for the first time that L-Arg supplementation may reduce interstitial fibrosis development in a mouse model of AAN. Mechanisms by which L-Arg exerts its protection against AA-induced nephrotoxicity remain speculative but some hypotheses can nevertheless be proposed. Until now, PTEC have always been considered as the primary target of AA-induced toxicity[8,9,47]. However, only few studies have addressed the involvement of the vascular network in AAN pathophysiology[4,12,16]. Nowadays, it is recognized in the literature that altered renal hemodynamics widely contribute to ongoing hypoxia and to the transition from AKI to CKD[28]. Therefore, we postulate that imbalance between endothelial vasoactive agents could contribute to renal dysfunction and, therefore, to fibrosis development in AAN. In this regard, L-Arg mediated protection should, at least in part, be due to restoration of NO deficiency thereby limits hypoxic injury to the kidney. Schneider et al[48] also proposed that L-Arg treatment could also overcome the accumulation of ADMA in their ischemic rat model. Moreover, protection could also come from additional non-hemodynamic cytoprotective effects. Indeed, L-Arg has already been shown to prevent the rise in lipid peroxides and the decrease in glutathione peroxidase activity, as induced in cyclosporine and in cisplatine treated rats[33,49]. We also previously reported that L-Arg reduces *Nox2* mRNA expression and ROS production in our AAN model, thereby decreasing AA-induced oxidative stress[10]. In this study, we confirmed that L-Arg decreases renal *Nox2* mRNA expression as well as *NRF-2* and *HO-1* mRNA expression (S3 Fig). Besides, the data obtained from our study may not exclude the possibility of the involvement of some L-Arg metabolites in this protective effect. In this regard, conversion to agmatine or stimulation of glucagon secretion could also be involved[50]. Nevertheless, we can reasonably confirm the involvement of NO considering the numerous studies reporting aggravated renal dysfunction and histopathological alterations after NO synthesis inhibition[33,35,36].

In conclusion, the present study brings further support to the role of NO in modulating nephrotoxicity and highlights the anti-inflammatory and anti-fibrotic properties of L-Arg supplementation in the evolution of AAN. The decrease in renal NO bioavailability contributes, at least in part, to the mechanism underlying the progression from AKI to CKD in AA-induced nephrotoxicity. Therefore, L-Arg seems to represent a promising agent in reducing renal inflammation and fibrosis occurring after acute or chronic intoxications.

## Supporting information

S1 Fig Comparison between control group and L-arginine group. (PDF)

S2 Fig Inflammatory and fibrosis parameters plotted on a fold-change scale (%). (PDF)

S3 Fig Effect of L-arginine supplementation on relative kidney expression of NADPH oxidase 2 (NOX2), NADPH oxidase 4 (NOX4), nuclear factor erythroid 2–related factor 2 (NRF2) and heme-oxygenase 1 (HO-1) mRNA (2-Δ ΔCT) at days 5, 10 and 20 in CTL, AA and AA+L-Arg mice. (PDF)
