# Supplementary material for: Restored nitric oxide bioavailability reduces the severity of acute-to-chronic transition in a mouse model of aristolochic acid nephropathy
Source: PLoS One. 2017 Aug 23;12(8):e0183604. doi: 10.1371/journal.pone.0183604 (PMC5568239; doi:10.1371/journal.pone.0183604)
Supplement: S1 Fig — (PDF) [file pone.0183604.s001.pdf]

S1 Fig. Comparison between control group (CTL) and L-Arginine group (L-Arg)

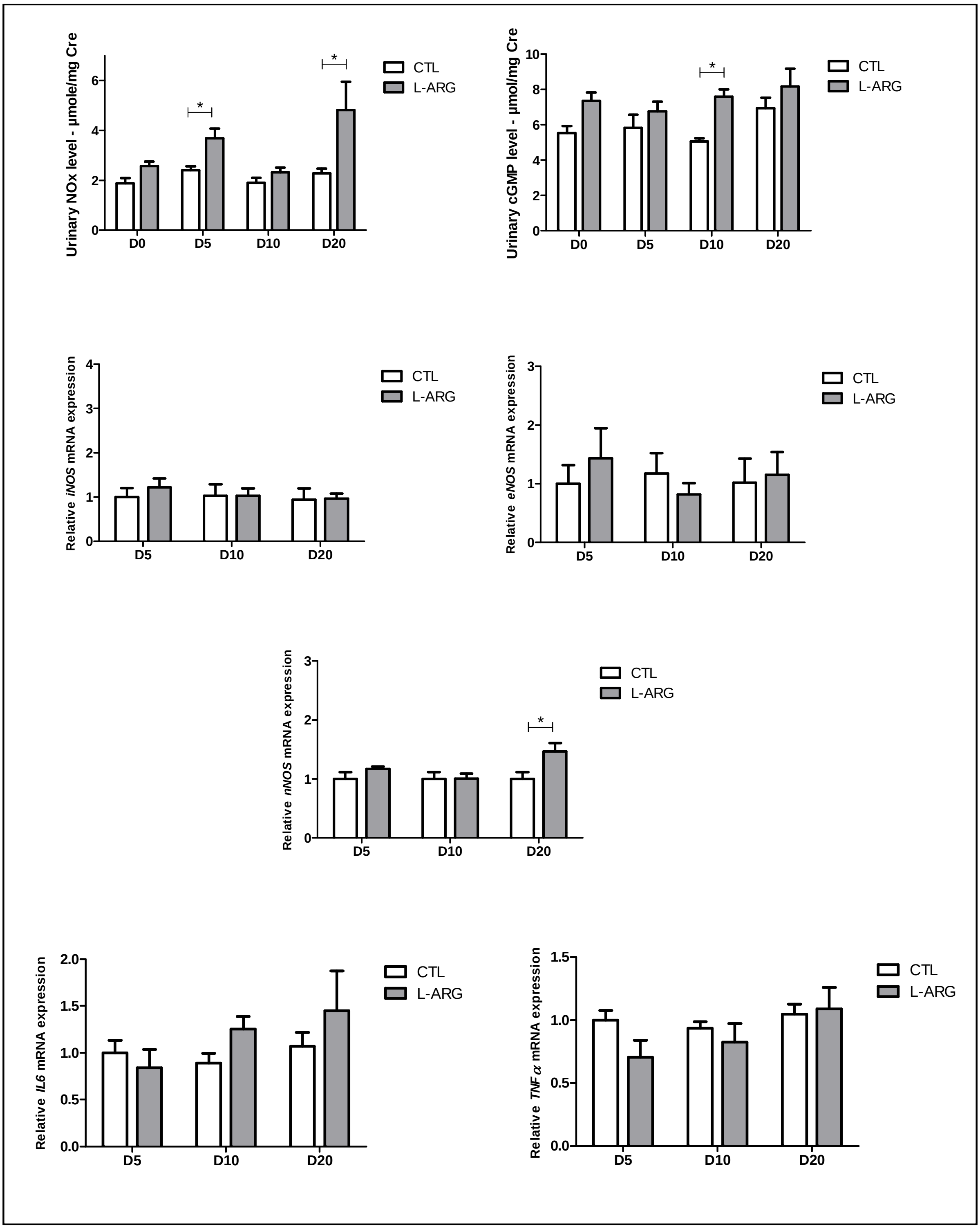

Statistical analysis were performed by two-way ANOVA followed by Holm-Sidak test. $^{*}P \leq 0{,}05$ *vs* CTL mice.
