# Supplementary figures and images for: Restored nitric oxide bioavailability reduces the severity of acute-to-chronic transition in a mouse model of aristolochic acid nephropathy
Source: PLoS One. 2017 Aug 23;12(8):e0183604. doi: 10.1371/journal.pone.0183604 (PMC5568239; doi:10.1371/journal.pone.0183604)

**S2 Fig. Inflammatory (A) and fibrosis (B) parameters plotted on a fold-change scale (%)**

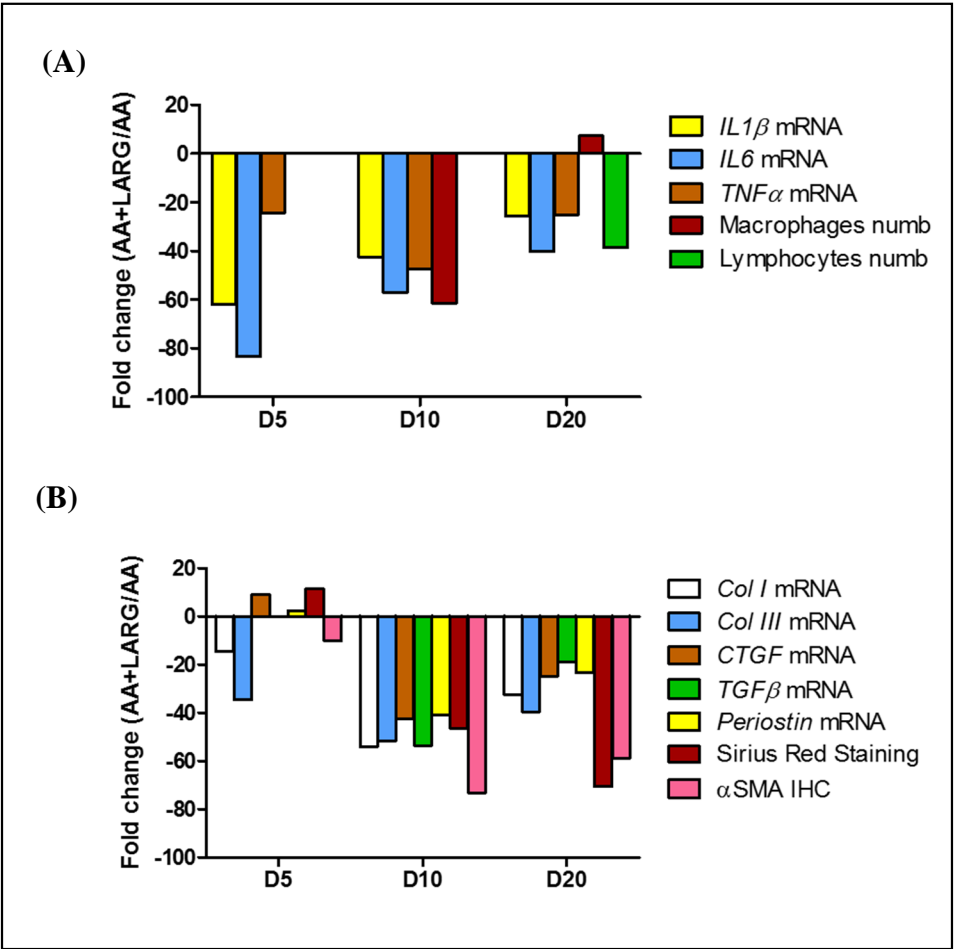

Supplement: S2 Fig — (PDF) [file pone.0183604.s002.pdf]
