# Supplementary material for: Restored nitric oxide bioavailability reduces the severity of acute-to-chronic transition in a mouse model of aristolochic acid nephropathy
Source: PLoS One. 2017 Aug 23;12(8):e0183604. doi: 10.1371/journal.pone.0183604 (PMC5568239; doi:10.1371/journal.pone.0183604)
Supplement: S3 Fig — (PDF) [file pone.0183604.s003.pdf]

S3 Fig. Effect of L-arginine supplementation on relative kidney expression of *NADPH oxidase 2* (*NOX2*), *NADPH oxidase 4* (*NOX4*), *nuclear factor erythroid 2–related factor 2* (*NRF2*) and *heme-oxygenase 1* (*HO-1*) mRNA ($2^{-\Delta\Delta CT}$) at days 5, 10 and 20 in CTL, AA and AA+L-Arg mice.

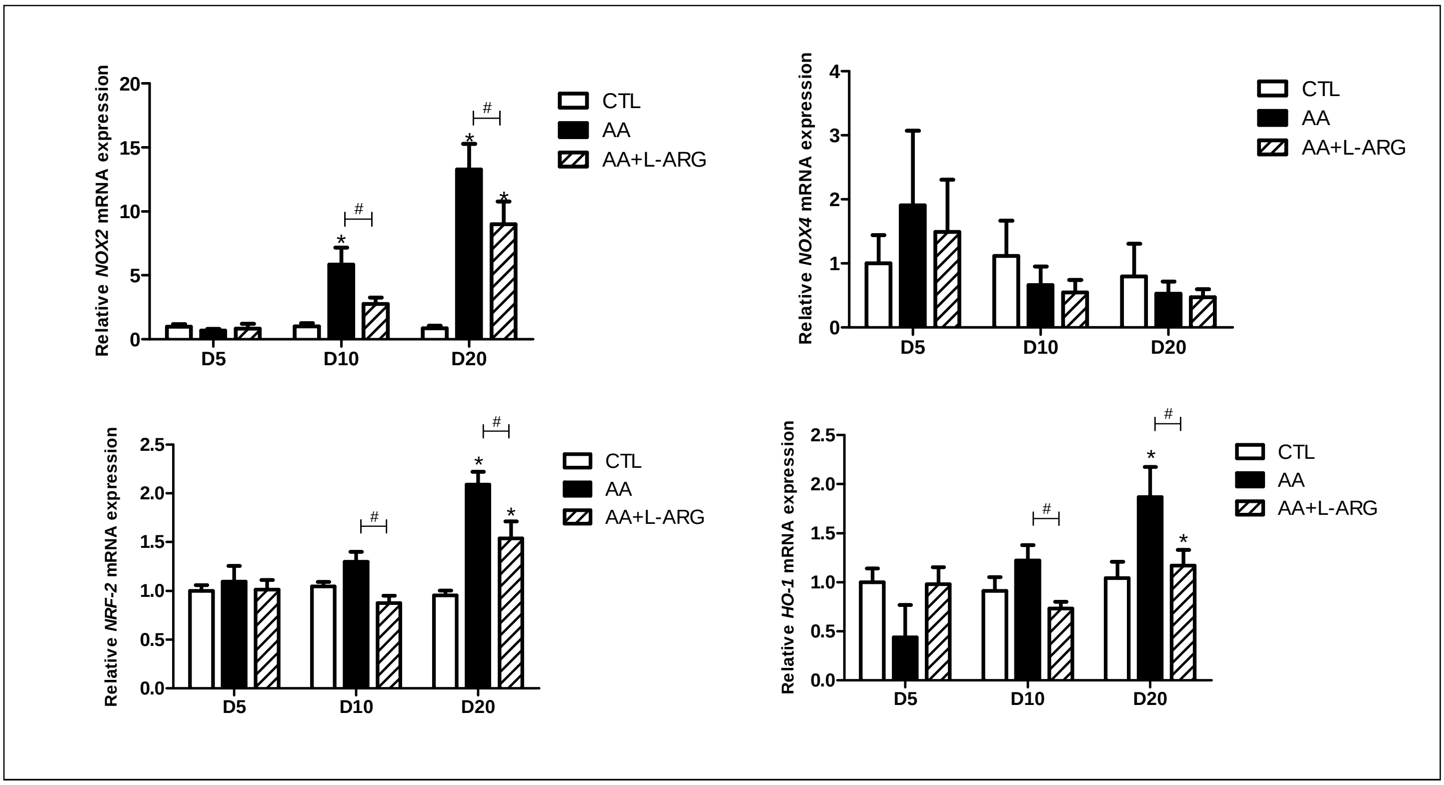

Statistical analysis were performed by two-way ANOVA followed by Holm-Sidak test. $^{*}P \leq 0{,}05$ *vs* CTL mice, $^{\#}P \leq 0{,}05$ *vs* AA mice.
